# Climate change‐induced migration patterns and extinction risks of Theaceae species in China

**DOI:** 10.1002/ece3.6202

**Published:** 2020-03-31

**Authors:** Yinbo Zhang, Qingxin Meng, Yuzhuo Wang, Xiaolong Zhang, Wei Wang

**Affiliations:** ^1^ College of Resources and Environment Shanxi University of Finance and Economics Taiyuan China; ^2^ College of Environmental and Resource Sciences Shanxi University Taiyuan China; ^3^ School of Ecological and Environmental Science & Tiantong National Station of Forest Ecosystem East China Normal University Shanghai China; ^4^ State Environmental Protection Key Laboratory of Regional Eco‐process and Function Assessment Chinese Research Academy of Environmental Sciences Beijing China

**Keywords:** biodiversity conservation, climate change, Red List, species distribution modeling, Theaceae species

## Abstract

Theaceae, an economically important angiosperm family, is widely distributed in tropical and subtropical forests in Asia. In China, Theaceae has particularly high abundances and endemism, comprising ~75% of the total genera and ~46% of the total species worldwide. Therefore, predicting the response of Theaceae species to climate change is vital. In this study, we collected distribution data for 200 wild Theaceae species in China, and predicted their distribution patterns under current and future climactic conditions by species distribution modeling (SDM). We revealed that Theaceae species richness is highest in southeastern China and on Hainan Island, reaching its highest value (137 species) in Fujian Province. According to the IUCN Red List criteria for assessing species threat levels under two dispersal assumptions (no dispersal and full dispersal), we evaluated the conservation status of all Theaceae species by calculating loss of suitable habitat under future climate scenarios. We predicted that nine additional species will become threatened due to climate change in the future; one species will be classified as critically endangered (CR), two as endangered (EN), and six as vulnerable (VU). Given their extinction risks associated with climate change, we recommended that these species be added to the Red List. Our investigation of migration patterns revealed regional differences in the number of emigrant, immigrant, and persistent species, indicating the need for targeted conservation strategies. Regions containing numerous emigrants are concentrated in Northern Taiwan and coastal regions of Zhejiang and Fujian provinces, while regions containing numerous immigrants include central Sichuan Province, the southeastern Tibet Autonomous Region, southwest Yunnan Province, northwest Sichuan Province, and the junction of Guangxi and Hunan provinces. Lastly, regions containing persistent species are widely distributed in southern China. Importantly, regions with high species turnover are located on the northern border of the entire Theaceae species distribution ranges owing to upwards migration; these regions are considered most sensitive to climate change and conservation planning should therefore be prioritized here. This study will contribute valuable information for reducing the negative impacts of climate change on Theaceae species, which will ultimately improve biodiversity conservation efficiency.

## Graphical abstract

Distribution patterns are predicted for 200 Theaceae species in China under current and future different climate change scenarios based on SDM and 11,781 occurrence records. Nine additional species are recommended to be added into Red List, due to their extinction risks to climate change. There are different migration patterns in the suitable distribution areas, so targeted protection strategies are needed. Regions with higher species turnover of migration are the most sensitive to climate change, which need to be given priority protection. The information developed here will contribute to reduce the negative impacts of climate change on Theaceae species.

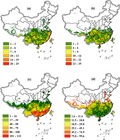

## 1 INTRODUCTION

Theaceae is an important angiosperm family that is widely distributed in tropical and subtropical regions of Asia (Min & Bartholomew, 2007). China harbors 12 genera and 274 species of Theaceae, encompassing ~75% of the total genera and ~46% of the total species worldwide. This family has extraordinarily high levels of endemism in China and contains the subfamilies Theoideae and Ternstroemioideae, which include two genera and 204 species endemic to China (Min & Zhang, 1996; Shen, Wu, Yang, Wang, & He, 2016). Additionally, Theaceae species have high economic value given that they include Chinese tea plants, edible and industrial oil plants, species used for construction, shipbuilding, and furniture (Shen et al., 2010), as well as medicinal and garden plants. Due to the high biological, cultural, and economic value of this group, it is important to conserve and sustainably manage wild Theaceae species, especially considering potential impacts of climate change on these species in the future.

The global climate is undergoing rapid changes toward generally warmer conditions. These changes are impacting overall biodiversity and more specifically, species distributions (Buytaert, Cuesta‐Camacho, & Tobón, 2011; Parmesan & Yohe, 2003). Reports from the Intergovernmental Science‐Policy Platform on Biodiversity and Ecosystem Services (IPBES) presented that the “distribution of 47% of the proportion of terrestrial flightless mammals and 23% of threatened birds may have already been negatively impacted by climate change, even for global warming of 1.5–2°C, the majority of terrestrial species ranges are projected to shrink profoundly” (IPBES, 2019). To track changing climates, species will need to shift their distributions to adapt to new environments, resulting in species migration, or, in extreme cases, extinction. In general, climate change forces species to migrate toward higher latitudes and upward in elevation (Parmesan, 2006; Robertj, David, Javier, & Victorj, 2007). In China, climate change will likely cause eastern forests to shift toward higher latitudes, temperate deciduous broad‐leaved forests to expand, and alpine meadows to retract upslope (Wang, Li, Huang, & Li, 2005; Zhao, Neilson, Yan, & Dong, 2002). Responses to climate change among Theaceae species in China will directly affect community composition and ecosystem services of subtropical evergreen broad‐leaved forests (Wang, 2006). To prescribe appropriate and actionable natural resource management strategies for these valuable species, it is imperative to determine the extent and direction of Theaceae species' range in response to climate change.

Previous studies of species within the Theaceae family have focused on their morphology, taxonomy, and molecular ecology (Gunathilake, Prince, & Whitlock, 2015; Li, Yang, Yang, & Li, 2011; Luna & Ochoterena, 2004). However, to our knowledge, no research has determined current or future ranges of this important plant family. Therefore, in this study, we proposed the following series of questions: How will climate change affected the overall distribution of Theaceae species in the future? Which species may be at greater risk of extinction under climate change conditions? In China, which direction and regions will Theaceae species likely migrate? Which conservation measures will be most effective in addressing and mitigating climate‐induced migration and extinction risks of Theaceae species in China?

To address these questions, we used species distribution modeling (SDM) to (a) predict the distribution patterns of wild Theaceae species under different current and future climate change scenarios, (b) evaluate species extinction risks based on loss of suitable habitat in the future, and (c) forecast migration patterns of Theaceae species under two dispersal assumptions (no dispersal and full dispersal). Our results aim to reveal the sensitivity of Theaceae species to climate change and will inform biodiversity conservation strategies seeking to facilitate species adaptation to climate change.

## 2 METHODS

### 2.1 Species data and environmental factors

We compiled a list of 274 Theaceae species according to the Flora of China (FOC; http://foc.eflora.cn/) and the Catalogue of Life China 2016 Annual Checklist (http://www.catalogueoflife.org/). We obtained species occurrences data from the National Specimen Information Infrastructure (NSII; http://www.nsii.org.cn/) and the Chinese Virtual Herbarium (CVH; http://www.cvh.ac.cn/). A total of 18,112 specimen records were collected and geo‐referenced according to the location descriptions provided on the labels. We collected records of accurate occurrences and distributions of wild Theaceae species, while ignoring distribution records of cultivated species. Occurrences were then overlaid onto 20 arc‐min grid cells (ca. 40 × 40 km), which resulted in 8,620 grid cells covering the entirety of China. We removed duplicate records of species from each cell, and species with occurrences in fewer than five cells were removed from the analysis. A total of 200 Theaceae species were retained, resulting in obtainment of 11,560 valid distribution records out of 12,055 original records of wild Theaceae species.

To model current and future responses to climate change, we selected 35 environmental factors, including 19 bioclimatic variables (1950–2000), 15 soil variables, and elevation. The elevation and bioclimatic variables were downloaded from WORLDCLIM (http://www.worldclim.org), and the soil variables were downloaded from the Food and Agricultural Organization (FAO, 2002). The spatial resolutions of these environmental factors were uniformly resampled to 20 arc‐min resolution. To avoid model over‐fitting caused by multi‐collinearity of variables (Graham, 2003; Pearson, Raxworthy, Nakamura, & Peterson, 2007), we performed Spearman's rank correlation tests to identify the least correlated environmental predictors (Spearman's rank correlation coefficient < 0.75) and removed them from the analysis; thus, we retained and analyzed the most important ecological predictors from groups of variables with correlations higher than 0.75. For the bioclimatic predictors (Table S1), we retained five variables: (a) Annual Mean Temperature (BIO01), (b) Isothermality (BIO03), (c) Annual Temperature Range (BIO07), (d) Annual Precipitation (BIO12), and (e) Precipitation Seasonality (BIO15). For the soil predictors (Table S2), we retained nine variables: (a) Base Saturation (%) Topsoil (BST); (b) CEC Soil Topsoil (CE‐S); (c) C: N Ratio Class Topsoil (CN‐T); (d) Organic Carbon Pool Topsoil (CP‐T); (e) Effective Soil Depth (Depth); (f) Soil Drainage Class (Drain); (g) Nitrogen (%) Topsoil (NN‐T); (h) Soil Production Index (Prod); and (i) Textural Class Subsoil (Text). In total, 15 predictors were used to model species' responses to climate, including five bioclimatic predictors, nine soil predictors, and elevation factor.

We based future projections on scenarios from the Intergovernmental Panel on Climate Change Fifth Assessment Report (IPCC, 2014) and chose a total of five scenarios: the General Circulation Model for the 2070s (BCC‐CSM‐1) and four emissions scenarios (RCP2.6, RCP4.5, RCP6.0, and RCP 8.5).

### 2.2 Species distribution modeling

Species distribution modeling allows construction of the correlative relationship between occurrence of a target species and environmental conditions, which can then be applied to the entire environmental space to predict the potential distribution of a species (Tsoar, Allouche, Steinitz, Rotem, & Kadmon, 2007). Maxent software has proven to perform well in modeling species distributions using algorithms based on presence‐only records and environmental data (Warren & Seifert, 2011), especially when attempting to model species distribution data that are not readily available (Graham et al., 2007; Zhang et al., 2013). In this study, we used Maxent software (ver. 3.3.1; http://www.cs.princeton.edu/~schapire/maxent/) (Phillips, Anderson, & Schapire, 2006) to predict current and future Theaceae species distribution. When conducting Maxent analysis, we set the following modeling rules: for species with 5–9 occurrence records, we used linear features; for species with 10 or more records, we used linear, quadratic, and hinge features (Raes & Steege, 2007). We assessed model accuracy using area under the curve (AUC) of the receiver operating characteristic (ROC) plot produced by Maxent.

Thresholds are required to project species distribution patterns in Maxent, which can convert continuous values to binary values (0, 1), representing species absence or presence in each grid cell. For species with 5–9 collection records, we used “equal training sensitivity and specificity” as the threshold (Liu, Berry, Dawson, & Pearson, 2005; Liu, White, & Newell, 2011, 2013). For species with 10 or more records, we used “conservative” fixed to “10 percent training presence” as the threshold (Raes, Roos, Slik, Loon, & Steege, 2009), which defines areas with a lower predicted value as species‐absent and those with a higher value as species‐present. After we set the thresholds, species distribution models of 200 Theaceae species were constructed resulting in five matrixes of distribution layers under the current climate scenario and four future emissions scenarios (RCP2.6; RCP4.5; RCP6.0; RCP8.5) in the 2070s, which are matrices of binary values (0, 1) or presence/absence data representing species distributions.

### 2.3 Distribution of species richness and weighted endemism

By superimposing the distribution patterns of 200 species under the current climate scenario, we generated the total distribution of Theaceae species in China. Further, we mapped the distribution of species richness by counting total species within each grid cell under different climate conditions.

In addition, we estimated patterns of endemism by calculating the weighted endemism index. This index weighs species richness according to the distribution range of various species, that is, the sum of the inverse of the distribution range of each species in each grid cell (Crisp, Laffan, Linder, & Monro, 2001; Kier & Barthlott, 2001; Küper, Sommer, Lovett, & Barthlott, 2010; Slatyer, Rosauer, & Lemckert, 2007). In this study, the range of species is the number of distribution grids. Thus, the distribution pattern of endemism was obtained by summing the weighted endemism index of all species distributions.

### 2.4 Extinction risk evaluation based on species habitat loss

Based on the obtained matrixes of presence/absence distribution predictions under current and future climactic conditions, we calculated the potential reduction of distribution ranges or loss of suitable habitat of species under two dispersal assumptions. The assumption of no dispersal does not allow for any range shifts, whereas the assumption of full dispersal allows for unrestricted species migration. Species were classified to different International Union for Conservation of Nature (IUCN) Red List categories based on habitat loss: Extinct (EX), Critically Endangered (CR), Endangered (EN), Vulnerable (VU), and Low Risk (LR) represent 100%, >80%, >50%, >30%, and <30% habitat loss, respectively (IUCN, 2001). Additionally, the most extreme climate change scenario was identified by comparing the magnitude of species extinction risks under different climate change scenarios, which can reveal the most severe potential threat to Theaceae in China.

### 2.5 Species migration patterns and sensitivity to climate change

Theaceae species migration patterns were determined under the previously identified most extreme climate change scenario and the assumption of full dispersal (Hole et al., 2011). We designated three types of species migration: (a) emigrant—species is present under current climactic conditions but absent under future conditions, (b) immigrant—species is currently absent but is present in the future, and (c) persistent—species is present under both current and future climactic conditions. Utilizing this approach, we obtained the distribution maps of emigrant, immigrant, and persistent species richness of Theaceae in China.

Furthermore, we introduced the “species turnover” variable, which combines migration data of emigrants, immigrants, and species richness to measure species composition changes in each grid cell (Zhang, Wang, Zhang, & Ma, 2014). The percentage of species turnover by pixel can be calculated according to the following formula from Thuiller, Lavorel, Araújo, Sykes, and Prentice (2005):

$$T=\left(I+E\right)/\left(SD+I\right)\times 100.$$

where *T* is the percentage of species turnover by pixel, *I* represents is the number of immigrants, *E* indicates the number of emigrants, and *SD* is the current species richness. Here, *T* can be used to evaluate species sensitivity to climate change. Therefore, grid cells with higher *T* values indicate larger changes in species composition and thus a higher sensitivity among those species to climate change.

## 3 RESULTS

**Figure 1:**
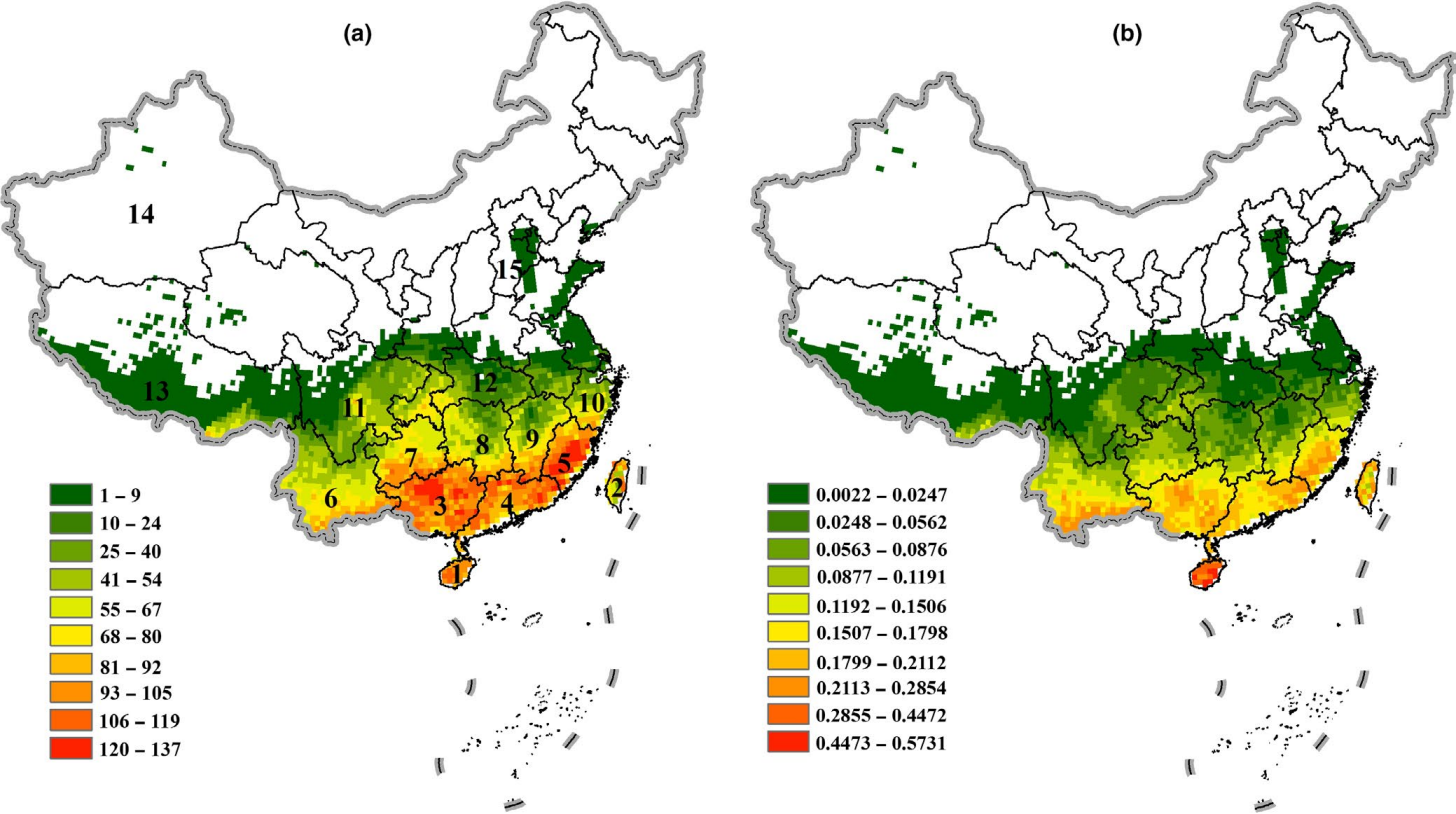
Theaceae species richness patterns in China under current climactic conditions. (a) Species richness; (b) Weighted endemism. Black numbers indicate administrative provinces: 1–Hainan, 2–Taiwan, 3–Guangxi, 4–Guangdong, 5–Fujian, 6–Yunnan, 7–Guizhou, 8–Hunan, 9–Jiangxi, 10–Zhejiang, 11–Sichuan, 12–Hubei, 13–Tibet, 14–Xinjiang, 15–Hebei

Our estimation of Theaceae species richness revealed that in China, Theaceae species are predominately distributed within tropical and subtropical regions, and richness gradually decreases as latitude increases (Figure 1). By classifying richness into ten intervals, we markedly revealed that Theaceae species are primarily located within the major regions of Southeastern China and Hainan Island. Fujian, Guangdong, Guangxi, and Hainan provinces have the highest species richness, containing over 120 Theaceae species. Fujian has the highest richness in all of China, harboring 137 species (Figure 1a). However, the distribution of species weighted endemism expands farther south than the distribution of species richness and high weighted endemism is only found in Hainan Province (Figure 1b).

**Figure 2:**
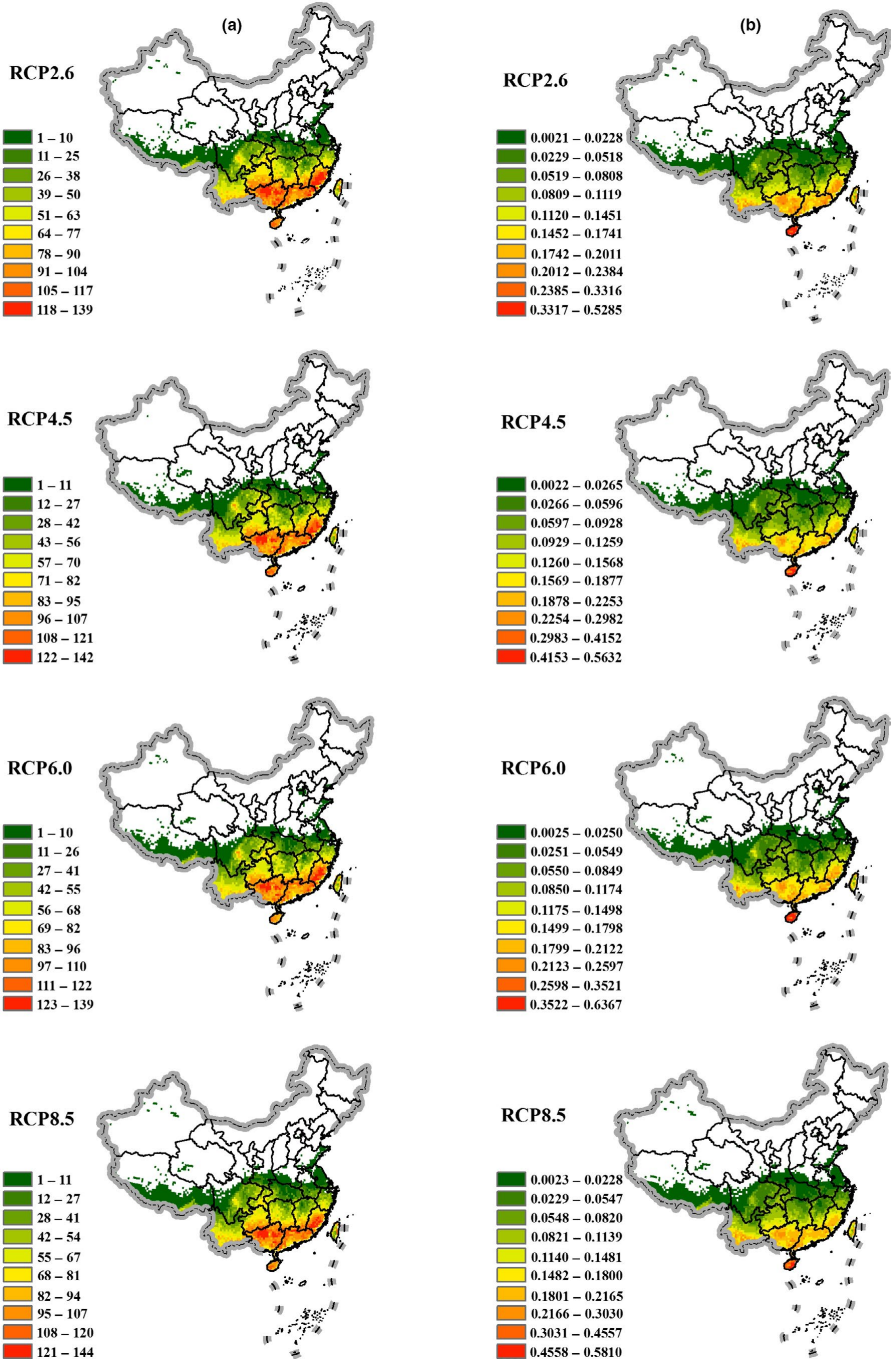
Theaceae species richness patterns in China under different climate change scenarios in the 2070s. (a) Species richness; (b) Weighted endemism

Overall, the distribution of species richness and weighted endemism visually exhibits a slight reduction in distribution ranges and shrinking in the northern regions of China compared with those distribution patterns under current and future climate scenarios (Figure 2). Further, distribution ranges under higher energy requirement scenarios will be reduced more than those under lower energy requirement scenarios.

According to the IUCN Red List criteria, we determined that several species of Theaceae will be under threat in the 2070s (Table 1), although they are currently classified as Least Concern (LC). Under the RCP6.0 emission scenario, more species become Critically Endangered (CR) than under other future scenarios. Therefore, we defined RCP6.0 as the most threatening future scenario to Theaceae in China. We proposed that nine species would need to be added to the Red List under this future scenario, resulting in 48 threatened Theaceae species (Table 2).

**Table 1:** Predicted extinction risks in the 2070s for Theaceae species in China

No dispersal assumption					Full dispersal assumption				
	LR	VU	EN	CR		LR	VU	EN	CR
RCP2.6	194	5	1	0	RCP2.6	196	4	0	0
RCP4.5	195	4	1	0	RCP4.5	197	3	0	0
RCP6.0	191	6	2	1	RCP6.0	194	4	1	1
RCP8.5	185	13	2	0	RCP8.5	194	5	1	0

Note

Predictions are based on four emissions scenarios and two dispersal assumptions. LR (Low Risk), <30% habitat loss; VU (Vulnerable), >30% habitat loss; EN (Endangered), >50% habitat loss; CR (Critically Endangered), >80% habitat loss.

**Table 2:** Red List of Theaceae species under current conditions and suggested species to be added in the future

Red list under current condition	CR	*Euryodendron excelsum* ^a^; *Eurya bifidostyla* ^a^
	EN	*Camellia candida* ^a^; *Camellia chrysanthoides* ^a^; *Eurya megatrichocarpa*; *Eurya wenshanensis* ^a^; *Ternstroemia hainanensis* ^a^; *Ternstroemia yunnanensis* ^a^; *Camellia parviflora* ^a^; *Eurya tsingpienensis*; *Camellia elongata* ^a^; *Camellia flavida* var. *flavida* ^a^; *Stewartia calcicola* ^a^; *Camellia melliana* ^a^
	VU	*Camellia szechuanensis* ^a^; *Cleyera obovata*; *Eurya persicifolia*; *Eurya taronensis* ^a^; *Apterosperma oblata* ^a^; *Camellia punctata* ^a^; *Camellia szemaoensis* ^a^; *Eurya polyneura*; *Schima villosa* ^a^; *Camellia ptilophylla* ^a^; *Pyrenaria sophiae* ^a^; *Camellia pachyandra* ^a^; *Camellia granthamiana* ^a^; *Ternstroemia conicocarpa* ^a^; *Camellia kwangsiensis* var. *kwangsiensis* ^a^; *Camellia villicarpa* ^a^; *Camellia assimiloides* ^a^; *Camellia crassipes* ^a^; *Camellia indochinensis* var. *indochinensis*; *Camellia crassicolumna* var. *crassicolumna* ^a^; *Eurya hupehensis* ^a^; *Camellia petelotii* var. *petelotii*; *Camellia taliensis*; *Camellia crapnelliana* ^a^; *Camellia reticulata* ^a^
Added Threatened species in the future	CR	*Adinandra nitida* ^a^
	EN	*Eurya lanciformis* ^a^; *Eurya emarginata*
	VU	*Eurya chukiangensis* ^a^; *Camellia hongkongensis* ^a^; *Adinandra megaphylla*; *Eurya metcalfiana* ^a^; *Eurya glandulosa* var. *glandulosa* ^a^; *Pyrenaria microcarpa* var. *microcarpa*

^a^ The species are endemic to China.

**Figure 3:**
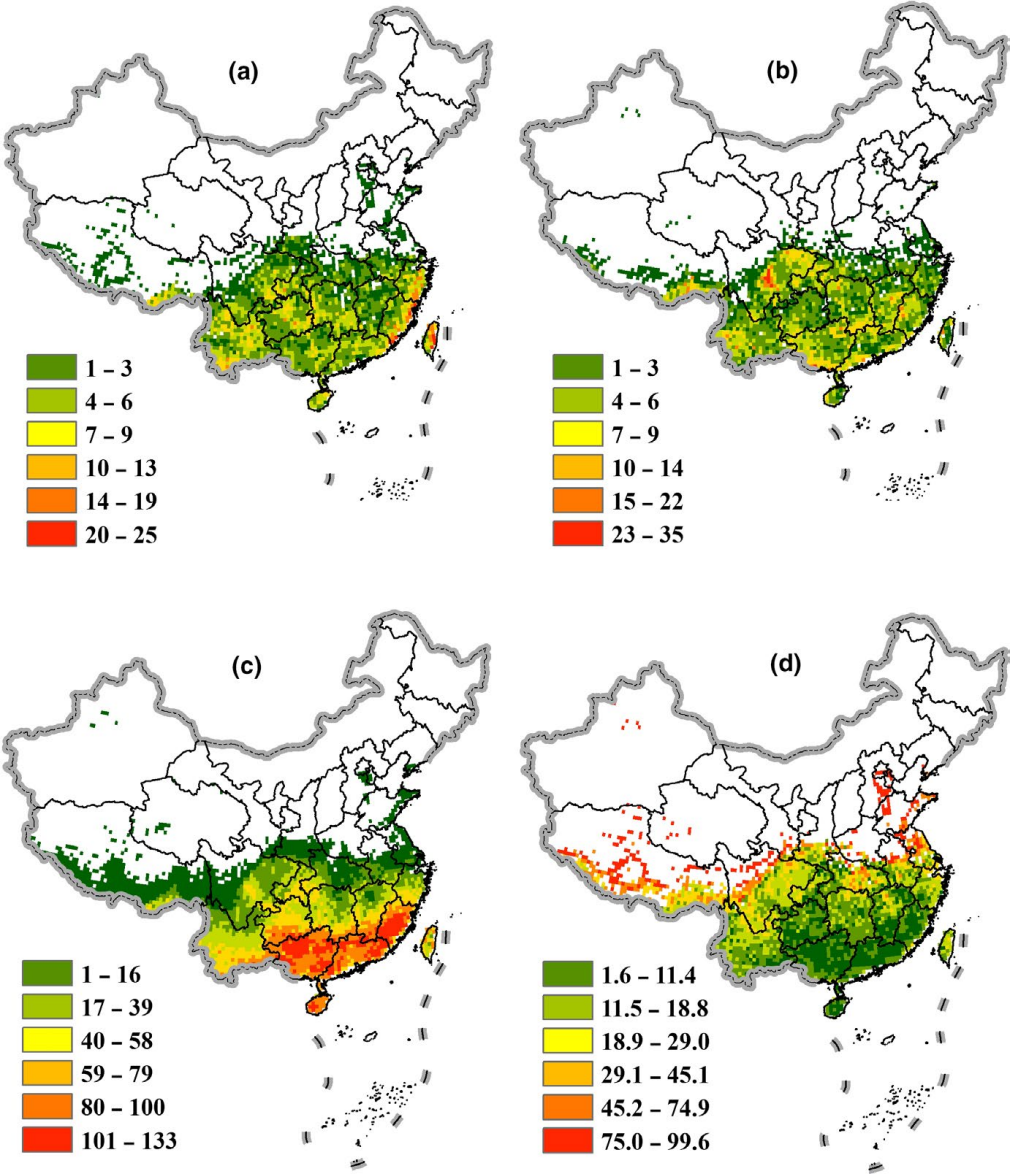
Migration patterns of Theaceae species in the 2070s under the most severe climate change scenario (RCP6.0). (a) Number of emigrants per grid cell; (b) Number of immigrants per grid cell; (c) Number of persistent species per grid cell; (d) Species turnover per grid cell

Under the most extreme climate scenario (RCP6.0), the migration types of Theaceae species differ in different geographic locations. Northern Taiwan and coastal regions of Zhejiang and Fujian provinces possess the highest number of emigrants (Figure 3a). Regions containing a high number of immigrants are located within idiosyncratic regions, including central Sichuan Province, the southeastern Tibet Autonomous Region, southwest Yunnan Province, northwest Sichuan Province, and the junction of Guangxi and Hunan provinces (Figure 3b). Persistent species are widely distributed in southern China (Figure 3c). Regions with high species turnover are primarily located on the northern border of the entire distribution range (Figure 3d). The northern border of Hubei Province has the highest species turnover, owing to high numbers of both emigrants and immigrants. The southern Tibet Autonomous Region and parts of Hebei Province further exhibit high species turnover driven mainly by emigration. Alternatively, higher species turnover in central Sichuan Province and the Xinjiang Autonomous Region is primarily attributable to immigration. Regions with significant species turnover, which represents drastic change in species composition, are considered the most sensitive to climate change.

## 4 DISCUSSION

### 4.1 Distribution of Theaceae species in China

China possesses the most diverse flora in the world, particularly in terms of seed plants and endemic woody seed plants, which are mainly distributed in mountainous areas of southern China (Huang et al., 2012; Ying, 2001). In this study, we determined that the greatest Theaceae species richness occurs in southeastern China and on Hainan Island, which is consistent with the distribution of subtropical evergreen broad‐leaved forest in China. In particular, regions containing the highest species richness occur in Fujian and Guangxi provinces, as suggested by Zhang et al. (2016).

In this study, we only predicted the distribution patterns of wild Theaceae species in China. As a plant family with high economic value, Theaceae distributions cannot be considered independently of human activity. Besides climate change, land‐use change and other human activities affect the location and extent of species. Thus, for individual Theaceae species with significant economic value, the potential variable of human influence should be effectively incorporated in future modeling efforts.

In recent years, the effects of climate warming on biodiversity distribution have received increasingly more attention (Bambach, Meza, Gilabert, & Miranda, 2013). In contrast to many studies which revealed that species shift their ranges toward higher latitudes and elevation in response to climate warming, we found that the overall distribution pattern of Theaceae taxa in China will not be significantly impacted by climate change. However, climate change impacts on individual Theaceae species cannot be ignored and must be further investigated in future research.

Overall, precipitation and temperature are the most important climate predictors and are therefore frequently analyzed in species distribution models (Bradie & Leung, 2017). For terrestrial plants, habitat characteristics also significantly influence species distribution, but these have been assessed less than precipitation and temperature. In our study, soil factors were also considered for the prediction of Theaceae species distribution, which effectively improved overall prediction accuracy, and our results confirmed that soil variables play a significant role in impacting species distribution. However, when predicting Theaceae distribution under future climate change scenarios, we only considered changes in climatic factors. Nonetheless, soil variable will undoubtedly be influenced under future climactic conditions, such as likely changes in CN‐T, NN‐T, CP‐T. Given the current lack of relevant soil prediction data, however, we only considered the impact of precipitation and temperature on species migration under future climactic conditions.

### 4.2 Species extinction risks and sensitivity to climate change

According to the China Biodiversity Red List (higher plants volume), there are 39 threatened Theaceae species with extinction risks, including two CR, 12 EN, and 25 VU (Table 2). By the 2070s, a total of nine additional Theaceae species will be threatened due to loss of >30% suitable habitat (Table 2). Although these nine species are not currently listed, we recommend their addition to the Red List to ensure implementation of appropriate conservation measures. Overall, according to the results of this study, there will be a new total of 48 threatened Theaceae species under extreme future climactic conditions, 37 of which are endemic to China.

Species migration or movement is an important ecological process that influences the prediction of species distributions, especially under global climate change (Holloway & Miller, 2017). Incorporating species movements into species distribution models would advance biogeographical and biological conservation of various species (Franklin, 2010). To explore extinction risks of Theaceae species under climate change, we incorporated dispersal assumptions (no dispersal and full dispersal) in predicting suitable future habitats for these species. Based on our predictions, we found that species extinction risks under the two dispersal assumptions are similar (Table 1). Further, the migrations of most Theaceae species are affected by climate change and not limited by dispersal capacity; thus, under future climactic conditions, only a small number of range shifts will be impacted by dispersal capacity. Similar conclusions were also noted in Yunnan's woody plant flora (Zhang et al., 2013). It is likely that latitudinal dispersal will not significantly contribute to species persistence in the future.

The ability of different species to migrate varies widely given that it is influenced by various important variables, including seed dispersal, interspecific relationships. These variables significantly influence successful species invasion and settlement in new habitats (Bradie & Leung, 2017). However, given that the current study was limited by a general lack of species data sources on the national scale, it can only reflect the impact of bioclimatic factors of climate change without consideration of species dispersal capacity and the method of plant propagation. We also neglected to study community dynamics that occur as a result of changes in intraspecific and interspecific relationships due to species migration or turnover, which is undoubtedly a complex and important issue (Mateo, Mokany, & Guisan, 2017). Thus, in future investigations, we suggest conducting more detailed simulations which consider more environmental factors at various scales (Han, Long, Fang, Hou, & Hong, 2019) to produce accurate extinction risk predictions and migration probability for individual species.

### 4.3 Theaceae species conservation in China

Potential climate change‐induced range shifts may reduce the effectiveness of existing biodiversity conservation strategies and protected area networks. Based on species migration patterns under climate change, conservation experts recommend flexible conservation planning strategies that account for predicted changes in habitat and dispersal capacity.

In regions with high numbers of emigrants, ex situ protection can be applied to facilitate Theaceae species' ability to cope with climate change. Seed banks and botanical gardens should be established for those species that will have a high likelihood of local extinction. It is not useful to establish new nature reserves in areas where emigrants will be locally extinct in the future. However, in regions with a high number of immigrants, effective land‐use planning is crucial for Theaceae conservation under climate change. Adequate protected areas and connective corridors are essential measures to preserve immigrants and facilitate migration. Furthermore, soil seed banks are essential to assist immigrants by providing seeds to establish new populations under future climactic condition. In most regions where Theaceae species will persist, climate change is not a significant threat. However, in situ protection is crucial for threatened Theaceae species under current and future climactic conditions.

Regions with high species turnover are not hotspots of emigration and immigration; nonetheless, emigration and immigration indeed occur within these regions which are also the most sensitive to climate change according to our results. These regions are primarily located on the northern edge of Theaceae distribution ranges, and climate change impacts will alter Theaceae distribution patterns and further affect the subtropical evergreen broad‐leaved forest ecosystem in these regions of China. To effectively conserve these regions, conservation planning should focus on optimizing land use and avoiding human‐induced habitat destruction. Combining in situ and ex situ protection measures would prove effective in strengthening the stability and elasticity of Theaceae species. These conservation recommendations will be useful to reduce the negative impact of climate change on Theaceae species in China.

## 5 CONCLUSIONS

Theaceae species play key roles in subtropical evergreen broad‐leaved forest ecosystems in China. Utilizing distribution data and species distribution modeling, we modeled patterns of richness and weighted endemism of Theaceae species in China. Models were then projected into the future to investigate suitable habitats under different climate change scenarios. We found that the overall distribution of Theaceae taxa in China will not be significantly impacted by climate change. However, we evaluated extinction risks and identified migration patterns under the most extreme climate change scenario (RCP6.0). This analysis revealed an additional nine Theaceae species will become threatened owing to loss of >30% suitable habitat in the future. Increased knowledge regarding migration responses of Theaceae species to climate change would improve conservation efficiency for these ecological and economically valuable species.

## CONFLICTS OF INTEREST

None declared.

## AUTHOR CONTRIBUTIONS

Wei Wang and Yinbo Zhang conceived and designed the experiment; Qingxin Meng and Yuzhuo Wang performed data collection and analysis; Yinbo Zhang wrote the paper; Xiaolong Zhang participated in the discussion of manuscript revision.

## Supporting information

TableS1‐S2

## Data Availability

Species distribution records of 200 Theaceae plants in China (XLS): Dryad https://doi.org/10.5061/dryad.tx95x69td.
