# Supplementary material for: Climate change‐induced migration patterns and extinction risks of Theaceae species in China
Source: Ecol Evol. 2020 Mar 31;10(10):4352–61. doi: 10.1002/ece3.6202 (PMC7246209; doi:10.1002/ece3.6202)
Supplement: Supplementary file 1 — TableS1‐S2 [file ECE3-10-4352-s001.docx]

Table S1 Spearman's rank correlation coefficients (r) for 5 bio-climate predictors; BIO1: Annual Mean Temperature; BIO3: Isothermality (BIO2/BIO7) (*100); BIO7: Temperature Annual Range (BIO5-BIO6); BIO12: Annual Precipitation; BIO15: Precipitation Seasonality (Coefficient of Variation).

|  | BIO01 | BIO03 | BIO07 | BIO12 | BIO15 |
| --- | --- | --- | --- | --- | --- |
| BIO01 |  |  |  |  |  |
| BIO03 | -0.127** |  |  |  |  |
| BIO07 | -0.429** | -0.506** |  |  |  |
| BIO12 | 0.528** | -0.131** | -0.621** |  |  |
| BIO15 | -0.572** | 0.174** | 0.376** | -0.396** |  |

Table S2 Spearman's rank correlation coefficients (r) for the 9 soil predictors; BS-T: Base Saturation% Topsoil; CE-S: CEC Soil Topsoil; CN-T: C: N Ratio Class Topsoil; CP-T: Organic Carbon Pool Topsoil; Depth: Effective Soil Depth; Drain: Soil Drainage Class; NN-T: Nitrogen% Topsoil; Prod: Soil Production Index; Text: Textural Class Topsoil.

|  | BS-T | CE-S | CN-T | CP-T | Depth | Drain | NN-T | Prod |
| --- | --- | --- | --- | --- | --- | --- | --- | --- |
| CE-S | 0.491** |  |  |  |  |  |  |  |
| CN-T | -0.023* | 0.249** |  |  |  |  |  |  |
| CP-T | 0.073** | 0.408** | 0.414** |  |  |  |  |  |
| Depth | 0.176** | -0.124** | -0.038** | 0.063** |  |  |  |  |
| Drain | 0.446** | 0.293** | 0.013** | 0.305** | 0.441** |  |  |  |
| NN-T | 0.279** | 0.526** | 0.328** | 0.588** | -0.14** | 0.183** |  |  |
| Prod | 0.521** | 0.187** | -0.235** | 0.165** | 0.422** | 0.614** | 0.098** |  |
| Text | 0.02** | 0.109** | 0.268** | 0.255** | 0.095** | 0.118** | 0.211** | 0.025** |
